# Prevalence of Viremic hepatitis C, hepatitis B, and HIV infection, and vaccination status among prisoners in Stockholm County

**DOI:** 10.1186/s12879-019-4581-3

**Published:** 2019-11-09

**Authors:** Caroline Gahrton, Gabriel Westman, Karin Lindahl, Fredrik Öhrn, Olav Dalgard, Christer Lidman, Lars-Håkan Nilsson, Karouk Said, Ann-Sofi Duberg, Soo Aleman

**Affiliations:** 1 Department of Infectious Diseases, Karolinska University Hospital, Huddinge, 141 86 Stockholm, Sweden; 2 Department of Medicine Huddinge, Karolinska Institutet, Stockholm, Sweden; 3 Department of Medical Sciences, Section of Infectious Diseases, Uppsala University, Uppsala, Sweden; 4 Center for Innovation, Karolinska University Hospital, Stockholm, Sweden; 5 Department of Infectious Diseases, Akershus University Hospital, Lørenskog, Norway; 6 Institute of Clinical Medicine, University of Oslo, Oslo, Norway; 7 The Swedish Prison and Probation Service, Norrköping, Sweden; 8 Department of Upper Gastrointestinal Diseases, Karolinska University Hospital, Stockholm, Sweden; 9 Department of Infectious Diseases, School of Medical Sciences, Örebro University, Örebro, Sweden

**Keywords:** Hepatitis C, Prevalence, Screening, Prison, HCV RNA, Viremic HCV infection, Hepatitis B, HIV, Vaccination

## Abstract

**Background:**

Identification and knowledge of settings with high prevalence of hepatitis C virus (HCV) infection is important when aiming for elimination of HCV. The primary aim of this study was to estimate the prevalence of viremic HCV infection among Swedish prisoners. Secondary aims were to estimate the prevalence of hepatitis B surface antigen (HBsAg), human immunodeficiency virus (HIV), and the proportion who have received hepatitis B virus (HBV) vaccination.

**Methods:**

A cross-sectional study of all incarcerated persons (*n* = 667) at all prisons (*n* = 9) in Stockholm County was conducted. All prisoners are routinely offered opt-in screening for HCV antibodies (anti-HCV), HCV RNA, HBsAg, anti-HBs, anti-HBc and HIV Ag/Ab at prison in Sweden. Data on the results of these tests and the number of received HBV vaccine doses were collected from the prison medical records. The parameters of HCV RNA, anti-HCV, and occurrence of testing for HCV were analysed in multiple logistic regression models in relation to age, sex and prison security class.

**Results:**

The median age was 35 (IQR 26–44) years, and 93.4% were men. Seventy-one percent (*n* = 471) had been tested for anti-HCV, 70% (*n* = 465) for HBsAg and 71% (*n* = 471) for HIV. The prevalence of anti-HCV, HCV RNA, HBsAg and HIV Ag/Ab was 17.0, 11.5, 1.9, and 0.2%, respectively among tested persons. The proportion of prisoners who had received full HBV vaccination was 40.6% (*n* = 271) among all study subjects.

**Conclusions:**

The prevalence of viremic HCV infection among Swedish prisoners in Stockholm County was 11.5%, which is high in comparison to the general population. Therefore, when aiming for the WHO goal of HCV elimination, prisons could suit as a platform for identification and treatment of HCV infection. There is a need to increase testing for blood-borne viruses and to improve vaccination coverage against HBV in Swedish prisons.

## Background

Patients with chronic hepatitis C virus (HCV) infection have increased risk of developing liver cirrosis and hepatocellular carcinoma [1, 2]. It is estimated that 71 million people (1.0%) have viremic HCV infection worldwide, and the corresponding number in Sweden is 41,000 or 0.4% of the population [3, 4].

Acute HCV infection occurs within the first 6 months after transmission of HCV, with 60–80% of infected persons developing chronic infection (i.e HCV infection more than 6 months) [5]. Viremic infection could consitute a chronic or acute infection.

The predominant route of infection in Western countries is injecting drug use. The prevalence of viremic HCV infection among people who inject drugs (PWID) is rather high in Sweden at approximately 65%, compared to other Western countries at 25–65% [6–8].

A history of injecting drug use is common among incarcerated persons [9]. Consequently, a high prevalence of HCV antibodies (anti-HCV) among prisoners globally has been reported, varying from 3 to 38% depending on region [10, 11]. The prevalence of viremic HCV infection has been less studied, with prevalence rates of 1.5–20% in a limited number of countries [12–22].

Among Swedish prisoners, 49% have used narcotics within 1 year before incarceration [23]. The most common crime of prisoners entering Swedish prisons 2017 was narcotic-related crime (30%) [23]. The prevalence of anti-HCV, indicating exposure to HCV, was estimated to 82% among PWID at the remand prisons in Stockholm during 1990–1998 [24]. It has prevously been speculated in e.g. the news media that the general prevalence of HCV in Swedish prisons could be 30–35% [25]. However, there is no published study determining the prevalence of anti-HCV or viremic HCV infection among incarcerated persons in Sweden.

Despite a high global prevalence of HCV among prisoners, a low proportion of these persons have been treated for HCV infection [26]. Interferon (IFN)-based treatment has previously been standard of care. This treatment is administered by subcutaneous injections and associated with poor cure rate of approximately 50%, frequent side effects, and a long duration of treatment often up to 1 year. Since 2014 new, oral, direct-acting antiviral treatment (DAA) has been introduced with > 95% cure rate, minimal side effects, and a treatment duration of only 8–16 weeks. In contrast to IFN-based treatment, DAA offers the possiblilty to treat patients with psychiatric co-morbidities, which are common among incarcerated persons [27].

Even though acute HCV can be spontaniously cleared, treatment of acute as well as chronic HCV infection, i.e. viremic infection, is recommended in European guidelines, and American guidelines suggests considering treatment of acute HCV when aiming to prevent HCV transmission [28, 29].

Hepatitis B virus (HBV) is transmitted through intravenous drug injection, sexual contact, mother to fetus, needle stick etc. Vaccination effectively prevents HBV infection [30] and was included in the infant vaccination program in all counties of Sweden from year 2016 [31]. The Public Health Agency of Sweden additionally recommends HBV vaccination to risk groups including PWID [31].

The WHO has called for elimination of hepatitis by 2030. To achieve this goal for HCV in past or present PWID, it is important to identify and target settings with high prevalence of viremic HCV infection, i.e. ongoing infection eligible for treatment, for example prisons. Correctional institutions could serve as an opportunity to engage HCV patients with otherwise little contact with health care providers. However, data on the prevalence of HCV in Swedish prisons is lacking. In this study we aimed at determining the prevalence of viremic HCV infection among incarcerated persons in Stockholm County. In addition, we aimed to estimate the prevalence of HBV and HIV infection, as well as the proportion who have received HBV vaccination.

## Methods

### Study subjects

This cross-sectional study included data from all incarcerated persons at all of the prisons (*n* = 9) in Stockholm County, Sweden. Data were collected from the medical records at seven prisons in May 2017 and two prisons in October 2017.

Eight of the prisons are for men and one is for women. Swedish prisons are divided into three security classes: 1, 2 and 3. Class 1 is the highest security class. The allocation of the prisoners to the classes depends on the risk of escape, criminal ties to other prisoners, and conviction. Among the nine prisons included, two prisons are of security class 1, four prisons of security class 2, and two prisons of security class 3. One prison is of both security class 2 and 3. As most of the incarcerated persons at this prison are in security class 2 and we did not have individual security class data, we labelled all persons at this prison as security class 2.

One nurse from the Swedish Prison and Probation Service (Kriminalvården) collected all the data for the study from the medical records at the prison facility. This nurse was the only person who had access to the personal identification data. Anonymized data was analysed by the researchers at Karolinska University Hospital/Karolinska Institutet.

### Virologic and vaccination data

In Sweden, all prisoners are routinely offered voluntary opt-in venous blood testing for HBV, HCV and HIV infection at the remand prison. A remand prison is a custody facility for persons who have been apprehended or arrested for a crime, waiting for trial or a place in prison. Also, HBV vaccination is offered. Opt-in means offering testing and conducting the test only in those that agree, as opposed to opt-out, which means that testing will be performed unless the person declines. If a person is sentenced to prison and has not accepted testing at the remand prison, the person will be offered testing when arriving to the prison.

A positive anti-HCV test is indicative of exposure to HCV, whereas viremic infection, i.e. ongoing infection, is defined as positive anti-HCV and HCV RNA, and could constitute either a chronic or acute HCV infection.

Active HBV infection is defined as positive hepatitis B surface antigen (HBsAg). Negative HBsAg combined with positive hepatitis B core antibody (anti-HBc) is indicative of past HBV infection. Prisoners, who accept HBV vaccination, are routinely given the first dose either at the time of testing for immunity against HBV, or afterwards. Therefore, the result of hepatitis B surface antibodies (anti-HBs) testing can be negative even though the prisoner is vaccinated. In order to estimate an accurate prevalence of vaccination coverage, we also registered if the person had been vaccinated for HBV in prison and the number of doses received.

For categorization of HBV immunity and vaccination, prisoners were divided into four groups: 1. Received full HBV vaccination (defined as either three doses of vaccine given in prison, or positive anti-HBs (if specified, > 10 IU/ml) combined with negative anti-HBc), 2. Exposed to HBV (defined as anti-HBc positive), 3. Susceptible to HBV infection (defined as not received three doses of vaccine in prison, combined with negative anti-HBs and anti-HBc), and 4. Potentially susceptible to HBV infection (defined as not tested and not received three doses of vaccine in prison).

HIV infection was defined as positive HIV antigen/antibody (Ag/Ab) test in combination.

The results of testing for anti-HCV, HCV RNA, HBsAg, anti-HBs, anti-HBc, and HIV Ag/Ab in combination was collected. If more than one test had been performed, the most recent was registered.

The analyses were routinely performed at local laboratories in Stockholm. Most analyses of anti-HCV, anti-HBc, HBsAg, anti-HBs, and HIV Ag/Ab were performed with Advia Centaur XP immunoassay system (Siemens Healthcare Diagnostics Inc., NY, USA). Most analyses of HCV RNA were performed with HCV RNA Cobas AmpliPrep/Cobas Taqman (Roche Diagnostics, Indianapolis, USA) before autumn 2017, and Aptima HCV RNA Quant Dx assay (Hologic, Toronto, Canada) from autumn 2017.

### Statistical analyses

The outcome parameters of HCV RNA, anti-HCV, and occurrence of testing for HCV in prison were analysed in three separate multiple logistic regression models in relation to age, sex and prison security class. The persons who were tested negative for anti-HCV and not tested for HCV RNA were assumed to be HCV RNA negative in the multiple logistic regression analyses. When estimating the prevalence of viremic HCV infection, the study population included all persons tested for anti-HCV with the exclusion of prisoners who were anti-HCV positive and not tested for HCV RNA. *P*-values below 0.05 were considered statistically significant. SPSS, version 25 (IBM Corp. in Armonk, NY) was used for all analyses.

## Results

### Participant characteristics

At the time of cross-sectional collection of data, 667 incarcerated persons were present at the nine prisons in Stockholm County and included in the study. The proportion of men were 93.4% (*n* = 623) (Table 1). The median age was 35 (Interquartile range, IQR 26–44) years for all persons, 35 (IQR 26.5–43.5) years for men, and 40 (IQR 25.6–54.4) years for women. The number of prisoners in security class 1, 2 and 3 were 293(44%), 281(42%), and 93(14%), respectively.

**Table 1:** Sex, age and security class of prisoners (*n* = 667) in Stockholm county

Characteristics, n (%)	
Sex	
Men	623 (93.4%)
Women	44 (6.6%)
Median age (IQR)	35 (26–44)
Security class	
1	293 (44%)
2	281 (42%)
3	93 (14%)

*Abbreviation*: *IQR* Interquartile range

### Prevalence of anti-HCV and viremic HCV infection

Of 667 study subjects, 471 (71%) had been tested for anti-HCV. Among the persons not tested (*n* = 196), the median age was 39.5 years and 179 (91%) persons were male. Prisoners in security class 2 and 3 were less likely to be tested for HCV compared to prisoners in security class 1 (*p* < 0.001) with adjusted Odds ratios (aOR) of 0.51 and 0.24, respectively (Table 3). Older persons were also less likely to be tested (aOR 0.98, *p* = 0.002). There was no significant difference in occurrence of testing for HCV between men and women.

**Fig. 1:**
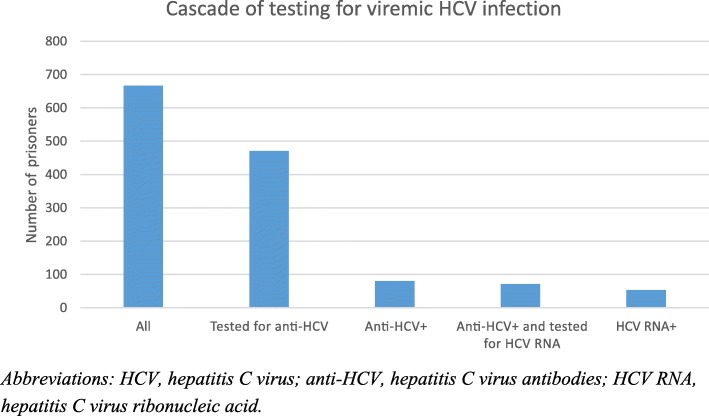
Cascade of testing for viremic hepatitis C virus (HCV) of incarcerated persons at all nine prisons in Stockholm County. *Abbreviations: HCV, hepatitis C virus; anti-HCV, hepatitis C virus antibodies; HCV RNA, hepatitis C virus ribonucleic acid*

Anti-HCV were detected in 80/471 (17, 95% CI 13.6–20.4%) persons (Table 2 and Fig. 1). Among the 80 anti-HCV positive persons, 71 were also tested for HCV RNA, whereof 53 (74.6, 95% CI 64.5–84.8%) were HCV RNA positive. All persons who were tested for HCV RNA had also been tested for anti-HCV, except one with missing anti-HCV data. This person was tested positive for HCV RNA in two different blood samples with 6 months interval and was therefore counted as positive, and thereby also categorized as “tested for anti-HCV”. Thus, the prevalence of persons with viremic infection (HCV RNA positive persons among tested for anti-HCV, with the exclusion of the nine persons not tested for HCV RNA among the anti-HCV positive) was 11.5% (95% CI 8.6–14.4%).

**Table 2:** Prevalence of hepatitis C (HCV), hepatitis B and HIV in all prisons (*n* = 9) in the Stockholm County

	All	Men	Women
Anti-HCV positivity, n^a^	80/471	71/444	9/27
(%; 95% CI)	(17.0; 13.6–20.4)	(16.0; 12.6–19.4)	(33.3; 15–6-51.1)
Viremic infection^b^			
HCV RNA+/anti-HCV+, n (%; 95% CI)	53/71 (74.6; 64.5–84.8)	50/63 (79.4; 69.3–89.4)	3/8 (37.5; 4.0–71.0)
HCV RNA+/tested for anti-HCV, n (%; 95% CI)	53/462 (11.5; 8.6–14.4)	50/436 (11.5; 8.5–14.4)	3/26 (11.5; 0.0–23.8)
Active HBV infection			
HBsAg+/tested for HBsAg, n (%; 95% CI)	9/465 (1.9; 0.7–3.2)	9/438 (2.1; 0.7–3.4)	0/27 (0)
HBsAg+/anti-HBc + , n (%; 95% CI)	9/65 (13.8; 5.4–22.2)	9/60 (15.0; 6.0–24.0)	0/5 (0)
Anti-HBc positivity			
Anti-HBc+/tested for anti-HBc, n (%; 95% CI)	65/440 (14.8; 11.5–18.1)	60/414 (14.5; 11.1–17.9)	5/26 (19.2; 4.1–34.4)
HIV infection			
HIV+/tested for HIV, n (%; 95% CI)	1/471 (0.2; 0.0–0.6)	1/444 (0.2; 0.0–0.7)	0/27 (0)

^a^Anti-HCV+/n tested for anti-HCV. One person who was not tested for anti-HCV was tested two times positive for HCV RNA with six months interval. This person was therefore counted as positive for anti-HCV and categorized as tested for anti-HCV

^b^Nine persons were not tested for HCV RNA among anti-HCV+ and excluded from analysis

*Abbreviations*: *Anti-HCV+* Hepatitis C virus antibody positive, *HCV RNA+* Hepatitis C virus RNA positive, *HBsAg+* Hepatitis B surface antigen positive, *Anti-HBc+* Hepatitis B core antibody positive, *HIV+* Human immunodeficiency virus positive, *CI* Confidence interval

The prevalence of viremic HCV infection as well as anti-HCV increased significantly with age (aOR 1.04 per year, *p* = 0.001, and aOR 1.05 per year, *p* < 0.001 respectively), but did not differ significantly between men and women, or between security classes (Table 3).

**Table 3:** Multiple logistic regression analyses of factors associated with occurrence of hepatitis C virus (HCV) testing, positive HCV antibodies, and viremic HCV infection

Predictor variable	Outcome variable		aOR (95% CI)	*P*-value
	Occurrence of HCV testing			
	Tested for anti-HCV *n* (%)	Not tested for anti-HCV *n* (%)		
Sex^a^				
Women, *n* = 44	27 (61.4)	17 (38.6)	1.00	
Men, *n* = 623	444 (71.3)	179 (28.7)	1.22 (0.62–2.40)	0.57
Age^a^ (per year)			0.98 (0.96–0.99)	0.002
Security class^a^				< 0.001
1, *n* = 293	238 (81.2)	55 (18.8)	1.00	
2, *n* = 281	189 (67.3)	92 (32.7)	0.51 (0.34–0.77)	
3, *n* = 93	44 (47.3)	49 (52.7)	0.24 (0.14–0.39)	
	Anti-HCV positivity			
	Anti-HCV+	Anti-HCV-		
Sex^b^				
Women, *n* = 27	9 (33.3)	18 (66.7)	1.00	
Men, *n* = 444	71 (16.0)	373 (84.0)	0.62 (0.24–1.61)	0.33
Age^b^ (per year)			1.05 (1.03–1.07)	< 0.001
Security class^b^				0.13
1, *n* = 238	37 (15.5)	201 (84.5)	1.00	
2, *n* = 189	39 (20.6)	150 (79.4)	1.21 (0.70–2.08)	
3, *n* = 44	4 (9.1)	40 (90.9)	0.38 (0.12–1.16)	
	Viremic infection			
	HCV RNA+	HCV RNA-		
Sex^c^				
Women, *n* = 26	3 (11.5)	23 (88.5)	1.00	
Men, *n* = 436	50 (11.5)	386 (88.5)	1.79 (0.47–6.85)	0.39
Age^c^ (per year)			1.04 (1.02–1.07)	0.001
Security class^c^				0.33
1, *n* = 235	24 (10.2)	211 (89.8)	1.00	
2, *n* = 183	25 (13.7)	158 (86.3)	1.41 (0.76–2.64)	
3, *n* = 44	4 (9.1)	40 (90.9)	0.66 (0.21–2.07)	

^a^Among all prisoners (*n* = 667)

^b^Among tested for anti-HCV (*n* = 471)

^c^Among tested for anti-HCV excluding nine persons who were not tested for HCV RNA among anti-HCV+ (*n* = 462)

*Abbreviations*: *HCV* Hepatitis C virus, *anti-HCV* Hepatitis C virus antibodies, *anti-HCV+* Hepatitis C virus antibodies positive, *HCV RNA+* Hepatitis C virus RNA positive, *HCV RNA-* Hepatitis C virus RNA negative, *aOR* adjusted odds ratio, *CI* Confidence interval

### Prevalence of HBV or HIV infection, and HBV vaccination status

Among the 667 persons 465 (70%) and 440 (66%) were tested for HBsAg and anti-HBc, respectively. Nine persons (1.9% of all tested, 95% CI 0.7–3.2%) were HBsAg positive. All HBsAg positive persons were also anti-HBc positive. Anti-HBc was positive in 65 persons (14.8% of tested, 95% CI 11.5–18.1%), including the HBsAg positive persons.

The prevalence of prisoners who had received full HBV vaccination was 40.6% (Table 4). Furthermore, 9.7% had been exposed and 49.6% were susceptible or potentially susceptible to HBV infection. Forty-eight persons without HBV exposure had been vaccinated with 1–2 doses in prison, but were not tested or negative for anti-HBs, and were thereby classified as potentially susceptible or susceptible. Six prisoners were anti-HBc positive and had nevertheless received three doses of vaccine in prison. These persons were categorized as exposed to HBV, as we presume that these persons had most likely received vaccination after exposure.

**Table 4:** Vaccination status among prisoners in Stockholm County

HBV vaccination status among all (*n* = 667) prisoners	
	N (%) of prisoners
Full HBV vaccination =3 doses of vaccine in prison or positive seromarkers for vaccination (anti-HBs positive + anti-HBc negative+ HBsAg negative)	271 (40.6)
Exposed to HBV = anti-HBc positive^a^	65 (9.7)
Susceptible to HBV = not received 3 doses of vaccine in prison combined with negative anti-HBs and negative anti-HBc^b^	124 (18.6)
Potentially susceptible to HBV = not received 3 doses in prison and not tested^b^	207 (31.0)

^a^including persons with active infection (HBsAg positive, *n* = 9) and persons immune due to previous infection (anti-HBc positive + HBsAg negative, *n* = 56)

^b^48 persons classified as susceptible or potentially susceptible had been vaccinated with 1–2 doses in prison

*Abbreviations*: *HBV* Hepatitis B virus, *anti-HBc* Hepatitis B core antibody, *anti-HBs* Hepatitis B surface antibody

Four hundred and seventy-one (71%) of all the 667 persons were tested for HIV and only one/471 (0.2% of tested, 95% CI 0.0–0.6%) was HIV positive.

## Discussion

In our study of 667 incarcerated persons at all prisons in Stockholm County, we found a high burden of HCV infection among prisoners, with an anti-HCV prevalence of 17% and a viremic prevalence of 11.5%. This is the first published study aiming at investigating these figures in Swedish prisons. The prevalence rates are considerably higher than in the general population, highlighting prisons as an important setting for diagnosis and treatment of HCV infection in Sweden [3].

The anti-HCV prevalence in our study is lower than a previously speculated prevalence of 30–35% in Swedish prisons [25]. This could indicate a decline in anti-HCV prevalence among incarcerated persons in Sweden in recent years, in line with the findings of a recent preliminary report of Danish prisoners, where a significant decrease in HCV prevalence within the last 20 years was reported [20]. The Danish study reported a concurrent increase in opioid substitution therapy (OST) coverage among prisoners during this time [20]. OST reduces drug dependence and injecting frequency and thereby the risk of HCV transmission among PWID [32]. The anti-HCV prevalence in our study is comparable to global estimates of 26 and 15% in two different previous studies [10, 33].

The prevalence of viremic HCV infection in our study is within the range of 1.5–20% that has been reported in a limited number of studies around the world, i.e. from Italy, Egypt, Brazil, Hungary, Spain, France, UK, Australia, USA, and Denmark [12–22].

Our study shows no significant difference in prevalence of positive anti-HCV or HCV RNA between men and women. A meta-analysis reported a higher anti-HCV prevalence among women, possibly due to a higher rate of incarceration of women for crimes associated with an increased risk of HCV [34]. In Sweden, the proportion of women sentenced for narcotic-related crimes and entering prison the year of 2017 was however similar to the proportion of the whole group of both men and women (30%), which may explain the absence of difference in our study [23]. Also, the proportion of women (38%) using narcotics the year before incarceration in Sweden, was even lower than among men (50%) [23]. In accordance with our result, a study of the Stockholm PWID population reported no substantial difference in prevalence of viremic HCV infection between men and women [6]. However, the lack of significant difference between men and women in our study could also be due to the low number of women included and not enough power to detect the difference.

The prevalence of both anti-HCV and HCV RNA increased with age, which is consistent with previous studies [11, 14, 18, 35]. However, older persons were less frequently tested in our study, suggesting that the true HCV prevalence of all prisoners could be even higher than our estimate. Therefore, emphasis on screening older prisoners should be made. On the other hand, it is also important to test younger prisoners, as younger PWID more often have active drug use with high risk of transmitting the disease [36].

In accordance with the increased HCV prevalence among the prisoners, the HBsAg and HIV prevalence of 1.9 and 0.2% in our study is higher than in the general Swedish population of 0.2 and 0.07%, respectively [37, 38]. Due to the higher disease burden of HBV and HIV infection in prison settings, strategies for prevention, screening, treatment, and linkage to care after release are important. For marginalized persons with drug use, psychiatric disorder, homelessness etcetera, incarceration could provide opportunity to access such care. The prevalence rates found in our study are lower than global estimates of 4.8% HBsAg and 3.8% HIV prevalence among prisoners, which correlates with a lower general HBsAg and HIV prevalence in Sweden than globally [33].

The vaccination coverage of 41% in our study is higher than numbers reported from prison studies in England and Spain, and in line with results from a French study [16, 17, 39]. A Scottish study reports increasing HBV vaccination uptake among PWID after the introduction of a universal HBV vaccination strategy in prison, and that vaccination was associated with reduced odds of HBV infection [40]. Vaccination is an easy and effective way of preventing HBV infection and the WHO recommends that all prisoners should be vaccinated against HBV [9]. Therefore, as 50% of the incarcerated persons in Stockholm County are susceptible or potentially susceptible to HBV infection, it is important to further increase the vaccination coverage.

A strength of this study is that all prisons in Stockholm County are included, which eliminates the risk of selection bias. Prisons in Stockholm County represent a substantial part (20%) of incarcerated persons in Sweden and frequently harbour persons from other parts of Sweden [23]. The median age of prisoners in all of Sweden 2018 was 34 years, 6% were women, and the proportion in security class 1, 2, and 3 were 32, 50, and 18% respectively [41]. Thus, the characteristics of the Swedish and Stockholm prison populations (Table 1) were similar. Therefore, the results found in this study may be generalizable to the incarcerated population in Sweden. However, further studies are required to confirm this result. If extrapolating our results to all prisoners in Sweden, we would expect approximately 480 persons to have viremic HCV infection in Swedish prisons at any point of time. This based on the fact that 4148 persons were incarcerated in Sweden at the time-point of 1st of October 2017 [23]. Up to approximately 1040 persons with viremic HCV infection could be present at Swedish prisons every year as 8000–9000 persons start a prison sentence each year in Sweden [23]. Another strength is that recall bias was avoided by using the medical records at the prison health care system as the source for data collection. An additional strength is that only one person collected all the data for the study, thereby avoiding inter-person variability.

A limitation of this study is that 30% of the prisoners have not been tested, introducing a potential selection bias. We found that older prisoners less often were tested, suggesting that the true prevalence could be somewhat higher. Another limitation is that we did not have data on confounding factors such as intravenous drug use, injecting risk behaviour, or OST treatment. An additional weakness is that the testing, from which the result was registered, was not only performed at the current incarceration but may have been performed at a previous sentence. Thus, some prisoners may have been infected in the period since last incarceration, resulting in a possible underestimation of the prevalence. On the other hand, some patients may have been treated since the last test was performed leading to a possible overestimation. We did not assess the uptake to HCV treatment in this study. However, as 20–40% of acute infections are spontaniously cured, the result of 75% viremic persons among those with anti-HCV in our study suggests that only few, if any, had received treatment [5]. Previous studies suggest that treatment of HCV infection in prison is feasible and cost-effective [42]. In Iceland, the prevalence of viremic HCV infection at prison decreased from 29 to 7% after the initiation of a nationwide treatment effort in 2016, which included an outreach nurse-led program within the penitentiary system. With the aim of HCV elimination, we suggest that treatment should be offered to all viremic prisoners during incarceration. Sweden does not yet have a national elimination plan for HCV. However, there is a plan to form a national hepatitis working group to work on an elimination plan in the fall of 2019 [43].

Varying rates of testing uptake (9–92%) in prisons in Western countries have been presented [44]. The WHO recommends that all prisoners should be tested for HCV [9]. Our result shows that there is a need for increased testing in prison. A strategy that has been reported to increase testing uptake is opt-out screening [45, 46]. In accordance, the Centers for Disease Control and Prevention recommends routine opt-out testing for HIV in correctional facilities [47]. The current strategy in Swedish prisons is opt-in testing, as in many European countries [48]. In order to increase testing uptake, we suggest that Swedish prisons should introduce opt-out screening for HCV, HBV and HIV. Similarly, to improve HBV vaccination coverage, we suggest that HBV vaccination should be offered with an opt-out approach.

Previous studies have reported continued injection drug use within the walls of the prison facilities, resulting in spread of HCV infection between prisoners. The incidence of HCV infection is reported to be 1.4 per 100 person-years among general prisoners and 16.4 per 100 person-years among prisoners with a history of injection drug use [10]. Our study did not address the question of new infections arising in prison. Future studies are needed to investigate this issue in Swedish prisons.

## Conclusions

Our study shows a prevalence of HCV viremia of 11.5% among Swedish prisoners, which is substantially higher than in the general Swedish population. With a high prevalence of HCV infected persons and the opportunity to provide new HCV therapies with facilitated treatment adherence in prison, we suggest that correctional institutions could suit as an excellent platform for identification and treatment of HCV infection in the efforts to achieve the WHO goal of global HCV elimination.

## Data Availability

The datasets used and/or analysed during the current study are available from the corresponding author on reasonable request.

## Abbreviations

Anti-HBc: Hepatitis B core antibody
C.I: Confidence interval
DAA: Direct-acting antiviral treatment
HBsAg: Hepatitis B surface antigen
HBV: Hepatitis B virus
HCV: Hepatitis C virus
Anti-HCV: Hepatitis C virus antibodies
HIV: Human immunodeficiency virus
IFN: Interferon
IQR: Interquartile range
OR: Odds ratio
PWID: People who inject drugs
RNA: Ribonucleic acid
